# *Linum lewisii* Adventitious and Hairy-Roots Cultures as Lignan Plant Factories

**DOI:** 10.3390/antiox11081526

**Published:** 2022-08-05

**Authors:** Roméo Arago Dougué Kentsop, Roberto Consonni, Michela Alfieri, Marina Laura, Gianluca Ottolina, Iride Mascheretti, Monica Mattana

**Affiliations:** 1 Institute of Agricultural Biology and Biotechnology, National Research Council, Via Bassini 15, 20133 Milan, Italy; 2 Institute of Chemical Sciences and Technologies “Giulio Natta”, National Research Council, Via Corti 12, 20133 Milan, Italy; 3 CREA Research Centre for Vegetable and Ornamental Crops (CREA OF), Corso degli Inglesi 508, 18038 Sanremo, Italy

**Keywords:** antioxidant capacity, elicitation, flax, justicidin B, lignans, precursor feeding, tissue cultures

## Abstract

Plants synthesize specific secondary metabolites for survival, reproduction, environmental resilience, and defense. Among them, lignans are a class of polyphenols with several bioactive properties: chemopreventive, anti-inflammatory, antiviral, and antioxidant. These compounds are often extracted from field-grown plants with very low yields. To overcome these constraints, in vitro tissue cultures provide a tool to optimize large-scale production. Moreover, the use of elicitation to increase secondary metabolite production is gaining importance. The aim of this work was to develop adventitious (ARL) and hairy roots (HRL) from *Linum lewisi*, a species able to synthesize arylnaphthalene lignans such as justicidin B. The ARL and HRL were obtained for the first time and characterized for their phenol content, antioxidant activity, and the production of justicidin B after treatments with several elicitors and precursor feeding. Through NMR spectroscopy, other four lignans were highlighted and identified in the roots extracts. A pilot-scale bioreactor was adopted to assess the suitability of the developed root cultures for future large-scale production. The ARL and HRL cultures showed a justicidin B production higher than other *Linum* species cultures described up to now (75.8 mg/L and 82.2 g/L), and the production more than doubled after elicitation with MeJA.

## 1. Introduction

Many plant secondary metabolites that occur ubiquitously in the plant kingdom are not directly involved in any physiological or metabolic response. However, they confer important ecological advantages to the plant attracting pollinators and repelling herbivores [1]. An interesting class of these molecules is constituted by lignans, a large group of dimeric phenylpropanoids widely distributed in higher plants [2]. Besides their involvement in plant defense mechanisms, lignans have numerous biological effects in mammals, including antitumor and antioxidant activities. Some plant lignans can be converted by intestinal microbiota to mammalian lignans (MLs), which may have protective effects against hormone-related diseases [3]. Chemopreventive properties and prevention of cardiovascular disorders, due to their antioxidant activity, have been demonstrated for MLs and lignans in general [4,5]. In this context, the demand for these compounds has increased in recent years.

The genus *Linum* comprises over 180 species, taxonomically divided into five sections forming two major clades [6]. The first clade (*Syllinum*, *Cathartolinum,* and *Linopsis* sections) accumulates a wide spectrum of aryltetralin lignans, such as podophyllotoxin [7,8]. This compound is of medical interest due to its strong cytotoxic activity, and it has been used as a precursor for semisynthetic antitumor therapeutics such as etoposide and teniposide [9,10]. Species belonging to the second clade (*Linum* and *Dasylinum* sections) accumulate mainly arylnaphthalene and aryldihydroarylnaphthalene lignans, such as justicidin B [7,8]. In the section *Linum*, justicidin B was isolated from *L. austriacum* [11], *L. lewisii*, *L. altaicum* [7], *L. narbonese*, *L. leonii* [12], and *L. glaucom* [13]. Justicidin B has cytotoxic, anti-inflammatory, antiviral, and antioxidant activity and inhibits platelet aggregation and bone resorption [14,15,16,17]. Thus, it may have significant clinical utility as a lead compound in the management of bone cancer and osteoclastogenesis [18,19,20].

In the last decades, challenging plant cell cultures treated with agents that mimic environmental insults have proved to be a successful strategy to increase the accumulation of bioactive compounds, such as lignans [21,22,23,24]. The main advantage of in vitro cultures over conventional whole plant cultivation lies in the possibility of obtaining the molecules of interest under controlled conditions regardless of climate change and soil characteristics throughout the plant cycle. Indeed, the cultures would be free of infections from microorganisms, and, even more important, the cells/tissues of any plants, particularly endangered or threatened plants, can easily be multiplied and manipulated to yield secondary metabolites [25,26]. The production of secondary metabolites is often low, less than 1% of plant dry weight, and represents a high metabolic cost to the plant. The biosynthetic machinery is present but not always active. Therefore, elicitation is a valuable strategy for improving the in vitro productivity of secondary metabolites.

Plant growth regulators, such as jasmonate derivatives, are the key components of plant defense response leading to the *de novo* transcription and translation that result in the enhancement of secondary metabolite biosynthesis [27]. When applied exogenously to the growth medium of in vitro cultures, these compounds could be effective elicitors. Methyl jasmonate (MeJA) is a plant-specific endogenous phytohormone involved in transducing external signals to activate defense reactions, including the reprogramming of metabolic pathways that initiate and enhance the production of defense compounds against herbivore insects and pathogens [28,29].

A molecular mimic of the isoleucine-combined form of jasmonic acid is coronatine (COR), an extremely potent elicitor-phytotoxin produced by the fermentation broth of *Pseudomonas syringae* pathovars [30,31]. Although the action of jasmonic acid or methyl jasmonate on secondary metabolite biosynthesis has been widely studied [32], there are relatively few reports on the effect of COR on secondary metabolite production [33].

Besides elicitor treatments, several organic compounds or biosynthetic precursors could be added to the culture medium to enhance the synthesis of secondary metabolites. This approach is known as precursor feeding, and it is useful when these molecules are inexpensive, and the biosynthetic pathway is even partially known. As an example feeding ferulic acid (FA) to cultures of *Vanilla planifolia* and *Decalepis hamiltonii* resulted in an increase in vanillin accumulation [34,35].

A further advantageous strategy is based on the simultaneous addition to the growth medium of two elicitors or the combination of elicitors with a precursor of the biosynthetic pathway of interest. It has been reported that root cultures of *Morus alba* co-treated with 200 µM MeJA and 2 mg/mL yeast extract accumulated higher contents of stilbenes compared with the yield obtained by adding only one of the two elicitors at a time [36]. Other authors observed that cell cultures of *Withania somnifera* treated simultaneously with squalene and chitosan greatly increased withanolides content compared to treatment with only one of the two compounds [37]. To enhance the production of psoralen in suspension cultures of *Psoralea corylifolia*, a strategy that combined MeJA and salicylic acid (SA) treatment with the psoralen biosynthesis pathway precursor trans-cinnamic acid was applied [38].

In this work, we developed adventitious and hairy root cultures from *L. lewisii* seedlings and compared the total phenol contents, the antioxidant activity, and the production of justicidin B among the root extracts subjected to different elicitor and/or precursor feeding treatments. To the best of our knowledge, this is the first time that in vitro cultures of *L. lewisii* have been employed for this purpose. Moreover, four other lignans have been highlighted, purified, and structurally characterized by the use of NMR spectroscopy [39].

In addition, at present, few efforts to produce bioactive molecules from large-scale cell cultures have been commercialized [40]. Regarding *Linum*, several attempts to scale up the production have been made, but none of them have yet led to the commercialization of these molecules from cell cultures [41,42]. In order to assess whether *L. lewisii* cultures could be suitable for future large-scale production, an explorative experiment using a pilot-scale bioreactor has been performed.

## 2. Materials and Methods

### 2.1. Plant Material and Cultures

*L. lewisii* seeds were obtained from Jelitto Perennial Seeds (https://www.jelitto.com, accessed on 1 February 2018). Seeds were sterilized in 70% (*v*/*v*) ethanol for 1 min, followed by sodium hypochlorite diluted 1:5 in water (*v*/*v*) and washed several times in sterile distilled water. Germination was obtained in phytatray (Merck, Darmstadt, Germany) on solid (0.8% *w/v* agar) Murashige and Skoog basal medium (MS, Duchefa, Haarlem, The Netherlands) at 22 °C in dark conditions. After one week, the seedlings were placed at 25 °C under 16 h light and 8 h darkness for one month.

To induce adventitious roots, leaves were collected from one-month-old plantlets of *L. lewisii*. The abaxial surface leaf explants were transferred to MS solid medium supplemented with 3% *w/v* sucrose, 0.4 mg/L 1-naphthaleneacetic (NAA) and 1 mg/L 2,4-dichlorophenoxyacetic acid (2,4-D). After 15–20 days of culture, roots primordia emerged. After four weeks, the roots of about 1.3 cm in length were individually transferred to an MS medium supplemented with 0.5 mg/L indole-3-butyric acid (IBA) and 0.1 mg/L indole-3-acetic acid (IAA) (MS-II medium), and they were subcultured every month. The adventitious root suspension culture (ARL) was started from 0.8 g fresh weight (FW) material in 50 mL of liquid MS-II medium. ARL growth was performed on an orbital shaker at 110 rpm at 25 °C in dark conditions.

The hairy roots formation was induced from leaf explants of in vitro seedlings of *L. lewisii* incubated with *Agrobacterium rhizogenes* strain ATCC 15,834 following the protocol described in Mascheretti et al. 2020 [41].

Seven independent hairy root lines were analyzed to verify the presence of the rolC gene of *A. rhizogenes*. For this purpose, the genomic DNA was extracted using DNeasy Plant Mini Kit (Qiagen, Hilden, Germany) according to the manufacturer’s instructions. The primer sequences used to amplify a fragment of the RolC gene were Forward: 5′-CGACCTGTGTTCTCTCTTTTTCAAGC-3′ and Reverse: 5′-GCACTCGCCATGCCTCACCAACTCACC-3′. The absence of *A. rhizogenes* DNA in the positive lines was verified by PCR amplification of the 326 bp fragment of virC1 following Vaira et al. [43].

All the media and components for in vitro cultures were purchased by Duchefa-Biochemie, Haarlem, The Netherlands.

### 2.2. Growth Measurement and Kinetics of HRL and ARL

The growth of *L. lewisii* cultures was measured as FW. Roots were harvested, washed three times with sterilized distilled water, dried on filter paper to remove all external moisture, and weighed. FW was expressed in grams/flask (g/flask). For the determination of root viability, a solution of 10 mM KH_2_PO_4_, 3% (*w*/*v*) sucrose and 0.25% (*w*/*v*) triphenyl tetrazolium chloride (TTC) was used as a visual indicator of root viability. To select the best performing HRL and ARL clones, respectively, each clone was inoculated in MS-II medium, and the biomass in each flask was determined after seven weeks of growth. The growth of the selected HRL and ARL clones was monitored for up to six weeks, and the FW was determined every week. The starting material was set at 0.5 g of fresh tissue, and each time point was replicated three times.

### 2.3. Elicitor and Precursor Feeding Treatments

Suspension cultures HRL and ARL were elicited with 100 μM MeJA (Merck, Darmstadt, Germany), 1 μM COR (Merck, Darmstadt, Germany), 50 μM and 100 μM SA (Merck, Darmstadt, Germany). MeJA was dissolved in absolute ethanol, COR, and SA in distilled water and sterilized by filtration (0.22 µm).

A total of 1 mM FA (Merck, Darmstadt, Germany) and 1 mM FA associated with 100 µM MeJA were used as precursor feeding of the suspension cultures. FA was dissolved in 0.07 N NaOH and sterilized by filtration (0.22 µm). The respective controls were performed by supplementing the medium with ethanol at the same final concentration or water. To verify the absorption of FA by the root cultures, the medium was collected at the end of the growing period, lyophilized, and resuspended in ethanol, sonicated for 15 min, then centrifuged and analyzed by HPLC. The separation was performed with a Kinetex 5 µm EVO C18 100 Å 250 × 4.6 mm at 35 °C, and the mobile phase was water +0.1% formic acid (solvent A) and acetonitrile +0.1% formic acid (solvent B). A solvent gradient at a flow rate of 1 mL/min was set as follows: 5% of B for 3 min, then a linear gradient up to 95% of B during 27 min, maintained for 5 min, and then re-equilibrated to the initial condition for 5 min. UV spectra were recorded from 200 to 400 nm with the registration of chromatogram at 320 nm.

Elicitor treatments and precursor feeding started on day 21 after the initiation of the liquid culture, and then the cultures were further incubated for seven days. The harvested roots were ground in liquid nitrogen, lyophilized, and stored at −80 °C until analysis.

### 2.4. Lignans Extraction

Lignans extraction was performed from powdered lyophilized tissues. The samples were dissolved in 80% (*v*/*v*) methanol and homogenized with Ika Ultra Turrax T18 for 2 min, vortexed, sonicated for 10 min, and extracted overnight on a gyratory shaker at room temperature in the dark. Extracts were then centrifuged for 20 min at 13.000× *g*, and the clear supernatants were used for total phenolics and flavonoids determination, and 1,1-diphenyl-2-picrylhydrazyl (DPPH, Merck, Darmstadt, Germany) radical scavenging assay and chromatographic analysis. For NMR analysis, the supernatants obtained after centrifugation were evaporated, and the solid residues were dissolved in deuterated chloroform (CDCl_3_) or deuterated methanol (CD_3_OD) according to their solubility.

### 2.5. Total Phenolic Contents and DPPH Radical Scavenging Activity

Total soluble phenolics were determined by Folin–Ciocalteu assay following the method of Ainsworth et al. [44]. The absorbance of the samples was spectrophotometrically measured at 765 nm using gallic acid (GA) as standard.

The amount of total flavonoids content was measured using quercetin as standard [43]. In detail, 50 μL of the sample diluted with 1.45 mL of water was mixed with 75 μL of 5% sodium nitrite and incubated for 5 min, followed by the addition of 150 μL of 10% aluminum chloride and further incubation of 6 min. Finally, 500 μL of 1 M NaOH was added, and the mixture was adjusted to 2.5 mL with water. The absorbance was spectrophotometrically measured at 425 nm. Total flavonoids content was expressed as milligrams of quercetin equivalents per gram of dry weight (mg QE/gDW).

The DPPH radicals scavenging assay was determined following the described method [44]. Briefly, a working solution of 0.208 mM DPPH in methanol was made daily and mixed with 30 µL of the extracts. The solution was incubated for 30 min at room temperature in dark conditions. The absorbance of the mixture was then spectrophotometrically measured at 515 nm against a blank of 80% (*v*/*v*) methanol. The ability to scavenge DPPH was calculated as indicated in Equation (1):

(%) DPPH radical scavenging activity = [(Act − Asa)/Act] × 100 (1)

where Act is the absorbance of DPPH radical plus methanol and Asa is that of DPPH radical plus the sample extract. Radical scavenging activity is shown as the percentage of DPPH inhibition/mg of DW (% inhibition DPPH/mgDW).

### 2.6. Chromatographic Analysis

The methanolic extracts were dried under nitrogen and then dissolved in chloroform. The obtained samples were qualitatively analyzed by thin layer chromatography (TLC) and quantitatively analyzed by high-performance liquid chromatography (HPLC).

TLC. The TLC chromatographic separation was performed following the method described by Romagnoli et al. [34]. Silica gel glass plates 60 (Merck, Darmstadt, Germany) 10 × 20 cm, 2 mm thickness were used for separation with a mobile phase composed of methanol–chloroform (1:99, *v*/*v*). The fluorescence at 366 nm was used for justicidin B identification [45]. The corresponding band was collected and resuspended in chloroform for subsequent analyses.

HPLC. HPLC analyses and purification were carried out as described in Mascheretti et al. [41].

### 2.7. NMR Structural Characterization of Extracts

NMR spectra were acquired by a 14.09 T Bruker DRX spectrometer operating at 600 MHz, equipped with a reverse z-gradient probe. All spectra were acquired at 300 K. The acquisition parameters for ^1^H experiments were as follows: monodimensional spectra have been recorded by acquiring 64 scans with 8000 Hz over 64 K data points, with a total relaxation time of 3.5 s. The solvent suppression scheme adopted for residual water suppression was based on low power irradiation with 36 µW, followed by four hard pulses of 8.06 µs each at 14.05 W. Bidimensional TOCSY (total correlation spectroscopy) was recorded by acquiring 64 scans, 256 experiments over 2 K data points and by using 90 ms of spin lock duration, and the other parameters as for 1D experiments. COSY-DQF (correlation spectroscopy-double quantum filtered) experiments were acquired with the same acquisition parameters of TOCSY, with a gradients ratio of 16/12/40, allowing the detection of double quantum coherences.

The conditions for ^13^C experiments were as follow: monodimensional spectra have been recorded by acquiring 8 K scans with 35,000 Hz of sweep width over 64 K data points, with a total relaxation time of 3 s. Additionally, for DEPT-135 (Distortionless Enhancement by Polarization Transfer) experiments, the direct ^1^H–^13^C coupling constant was set to 145 Hz, and 512 scans were acquired. Bidimensional edited HSQC (Heteronuclear Single Quantum Coherence) and HMBC (Heteronuclear Multiple Bond Correlation) were acquired by using 256 scans and 256 experiments over 2 K data points, with 145 Hz and 8 Hz for direct and long-range heteronucler coupling constants, respectively. Sweep widths were set to 8000 Hz and 35,000 Hz for ^1^H and ^13^C, respectively.

NMR data were acquired and processed by using TOPSPIN software (Bruker, v.3.1), and spectra were referenced to the solvent signals (chloroform at 7.27 ppm and methanol, 3.31 ppm).

### 2.8. Roots Cultivation in Bioreactor System

HRc and ARc were cultured for 4 weeks in a 1 L stirred tank bioreactor (Infors HT Labfors, Bottmingen-Basel, Switzerland) containing 650 mL of fresh free-hormones MS medium. The elicitated samples were grown for three weeks, elicited with 100 μM MeJA and grown for a further one week. The starting material was approximately 14 g of FW of roots.

The vessel of the bioreactor was modified with a plastic mesh as root support and separate from the stirring blades. The bioreactor was maintained at 25 °C in the dark and aerated with sterile air at 80 mL/min. The fluid mixing was carried out at 50 rpm. The pH of the medium and the percentage of dissolved oxygen were continuously monitored.

The lignan extraction was performed according to [41].

## 3. Results

### 3.1. Adventitious Roots and Hairy Root Cultures Lines Analysis and Selection

**Figure 1:**
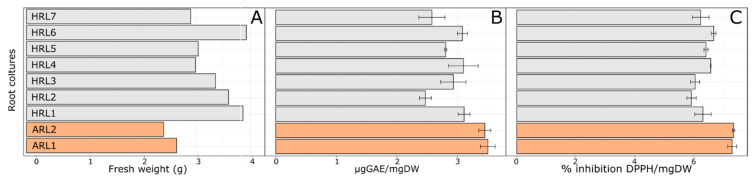
(**A**) Biomass of *L. lewisii* hairy and adventitious root cultures expressed as gram (g) of FW per flask; (**B**) total phenol content expressed as µg GAE/mgDW; (**C**) radical scavenging activity expressed as % inhibition DPPH/mgDW. From HRL1 to HRL7: hairy root lines, ARL1 and ARL2: adventitious root lines. The analyses were performed on roots extracts after six weeks of growth. Each bar represents the average of three biological replicates ± SD.

Seven different lines of hairy roots (from HRL1 to HRL7) and two adventitious roots (ARL1 and ARL2) were obtained from *L. lewisii* seedlings. With the aim to evaluate the best performing HRL and ARL lines to be used in the experiments hereafter, 0.5 g of starting fresh material were inoculated in a liquid medium. After six weeks of growth, the biomass, the total phenolic contents, and the antioxidant activity of the extracts were evaluated (Figure 1).

Overall, the growth performance of HRL lines was better than ARL lines (Figure 1A,B). Nevertheless, after six weeks of growth, all the analyzed clones appeared healthy with well-developed lateral branching, and no signs of browning were present. Specifically, within each group, HRL6 and ARL1 reached the highest biomass at the end of the experiment. On the contrary, total phenols accumulation was significantly higher in the ARL group than in HRL (Figure 1B). Conversely, radical scavenging activity showed a similar trend among the two groups (Figure 1C). In particular, antioxidant capacity was higher in the ARL group than in HRL, reaching in ARL1 a value of 7.35% inhibition DPPH/mgDW.

Based on these results, HRL6 and ARL1 lines have been chosen for all the following experiments.

### 3.2. Growth Kinetic of HRL6 and ARL1 and Accumulation of Justicidin B

**Figure 2:**
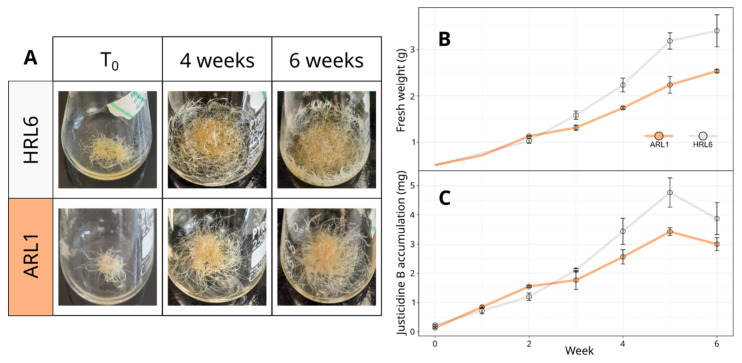
(**A**) Picture of HRL6 and ARL1 cultures at 0, 4, and 6 weeks. (**B**) Growth kinetic of *Linum lewisii* HRL6 and ARL1 cultures. FW: fresh weight expressed as grams (g) per flask. Each value represents the average of three biological replicates ± SD. Student’s *t*-test was applied. (**C**) Accumulation of justicidin B in HRL6 and ARL1 extracts expressed as mg of justicidin B.

Since secondary metabolites are mostly synthetized during the exponential phase [32,33], the elicitor treatments and/or precursor feeding were applied to root cultures just entered in this period. Therefore, the growth kinetic of HRL6 and ARL1 cultures was evaluated as FW in a time-course experiment lasting six weeks. In Figure 2A the differences between the two root cultures are reported. In Figure 2B, the growth curve of HRL6 showed a lag period lasting two weeks, and then an exponential phase occurred until the fifth week. Thereafter, the culture entered a stationary phase. Similar behavior was observed for ARL1, although the growth in the exponential phase was less marked than for HRL6; therefore, the biomass reached by ARL1 at the end of the stationary phase (week 5) was significantly lower than HRL6. In particular, HRL6 displayed a significance versus ARL1 at 4 (*p* ≤ 0.05), 5, and 6 weeks (*p* ≤ 0.01).

Concurrently, the amount of justicidin B in root samples collected each week was evaluated by HPLC, generating a curve of temporal accumulation of this metabolite in *L. lewisii* root cultures (Figure 2C). The content of justicidin B, expressed as total mgs, was higher in HRL6 at each time point except after the first week, where the content of justicidin B in the two-line extracts was similar. The justicidin B content in HRL6 was significantly higher than ARL1 at 3, 4, and 5 weeks (*p* ≤ 0.05). The amount of the molecule showed the maximum storage after 5 weeks of growth in both roots lines (4.77 ± 0.50 and 3.43 ± 0.14 mg in HRL6 and ARL1, respectively). HPLC analysis on methanolic extracts of HRL6 and ARL1 revealed the presence of other molecules besides justicidin B.

### 3.3. NMR Structural Characterization of HRL6 and ARL1 Extracts

**Figure 3:**
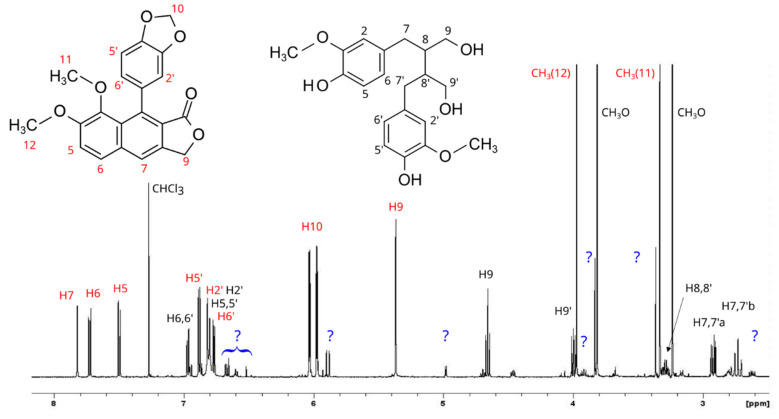
^1^H NMR spectrum of isolated isojusticidin B (**left**) containing traces of *secoisolariciresinol* (**right**). The blue marks belong to unassigned compounds.

In addition to justicidin B, some of the compounds revealed by the HPLC separation of the HR and AR extracts were purified and then studied by NMR. The spectrum of the first isolated compound showed the presence of dominant signals due to arylnaphtalene lignan and other signals due to unknown molecules in a lower amount, Figure 3. The aromatic region of the spectrum revealed the presence of three aromatic protons belonging to the typical naphthalene moiety. Using these protons as entry points for the resonance assignment strategy, the combined use of HSQC and HMBC allowed us to unambiguously identify this molecule as isojusticidin B. The presence of the other two methyl groups’ resonances in the ^1^H NMR spectrum suggested the concomitant occurrence of another structure with an aromatic ring carrying the two methyl groups. In addition, in this case, the combined use of HSQC and HMBC experiments allowed us to assign the signals to *secoisolariciresinol*. A third molecule, whose presence was very low, was detected by the resonances of two methyls bound to an aromatic ring and was not elucidated (Figure 3).

**Figure 4:**
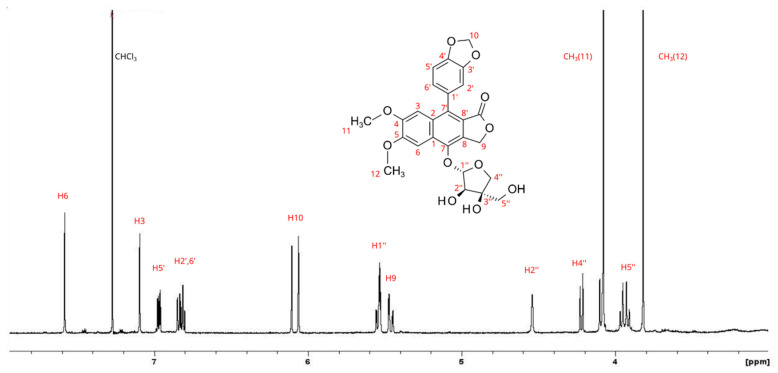
^1^H NMR spectrum of tuberculatin.

The dry extract of the second compound purified was dissolved in deuterated chloroform. The ^1^H NMR spectrum represented in Figure 4 showed the presence of a single molecule still belonging to the arylnaphtalene lignan moiety. The single aromatic signal, the resonances of two methyls, and the signal at 5.53 ppm partially overlapped with other signals suggested the structure of a glycosylated arylnaphtalene lignan. The combined use of HSQC and HMBC allowed the full characterization of the molecule as 7-*O*-β-d-apiofuranosyl-diphyllin, also known as tuberculatin.

**Figure 5:**
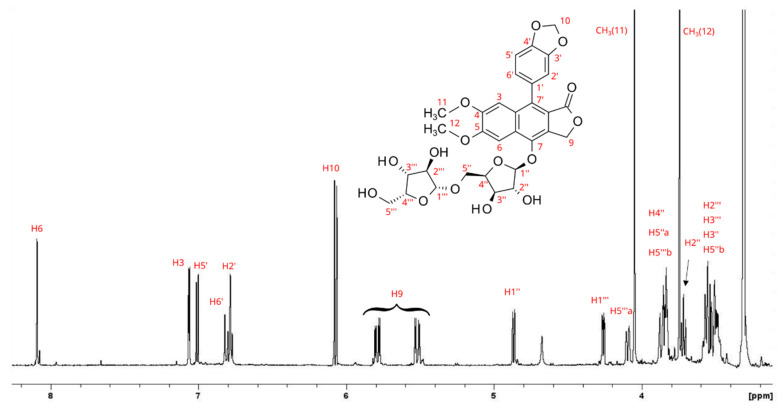
^1^H NMR spectrum of 7-*O*-β-d-xylofuranosyl-(1→5)-*O*-β-d-xylofuranosyl-diphyllin.

The presence of justicidine B was confirmed by NMR as already described [41]. It is interesting to note the absence of diphyllin as it is, but as the glycosylated forms, which were soluble only in methanol. As observed in Figure 5, the aromatic region revealed the presence of five aromatic protons. Additionally, two methyl groups were present, and another two anomeric signals belonging to a glycosyl moiety were identified. The aglycone moiety was rapidly identified as arylnaphthalene lignan, in particular to the glycosylated diphyllin moiety. The identification of the glycosylic moieties required extensive NMR characterization, employing several bidimensional heteronuclear experiments. In detail, the DEPT-135 experiment allowed for evaluating the presence of 12 carbon atoms belonging to saccharide structure, including two CH_2_ groups, while from the ^1^H NMR spectrum, the integral ratio confirmed the presence of 12 saccharide protons. The HMBC spectrum readily revealed a long-range ^1^H–^13^C correlation between the anomeric proton signal at 4.87 ppm with carbon C7 (146.1 ppm) of diphyllin. The combined use of -COSY-DQF, DEPT-135, and edited HSQC HMBC spectra (Figures S1–S4) allowed us to define the full assignment of the two saccharide moieties, whose both ^1^H and ^13^C signals and correlations were fully compatible with the represented structure, namely 7-*O*-β-d-xylofuranosyl-(1→5)-*O*-β-d-xylofuranosyl-diphyllin and observed for the first time.

**Figure 6:**
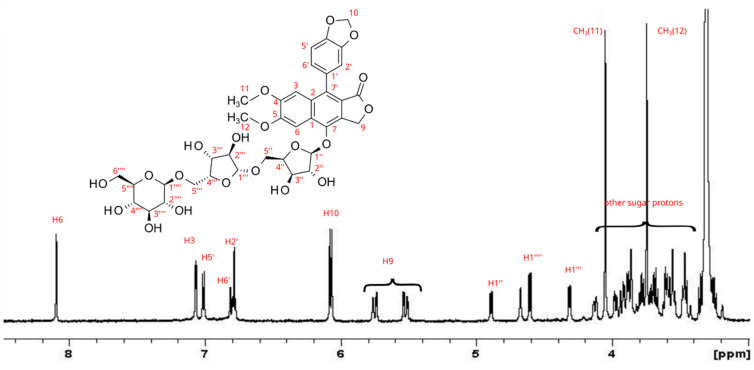
^1^H NMR spectrum of 7-*O*-β-d-xylofuranosyl-(1→5)-*O*-β-d-xylofuranosyl-(1→5)-*O*-β-d-glucosyl-diphyllin.

A companion new compound was identified as 7-*O*-β-d-xylofuranosyl-(1→5)-*O*-β-d-xylofuranosyl-(1→5)-*O*-β-d-glucosyl-diphyllin. The ^1^H NMR spectrum (Figure 6) clearly showed the aglicone moiety with a diphyllin structure, with an additional glycosylic moiety, whose anomeric proton occurred at 4.61 ppm (H_1_^’’’^ in Figure 6). In addition, in this case, the combined use of COSY-DQF, DEPT-135, edited HSQC, and HMBC spectra (Figures S5–S8) allowed us to identify the connection of anomeric proton of glucose to the H_5_^’’^ of the second xylofuranosyl residue, thus defining the structure determination.

### 3.4. Effects of Elicitors and/or Precursor Feeding Treatments on Phenols and Antioxidant Activity

Based on the growth kinetics of root cultures (Figure 2A), the elicitor treatments started 21 days after the inoculum of the roots in a liquid medium; then, the cultures were allowed to grow for a further 7 days. Before starting the treatment, the percentage of roots viability was evaluated, and an 80–90% rate of success was considered acceptable to proceed with the experiments.

**Figure 7:**
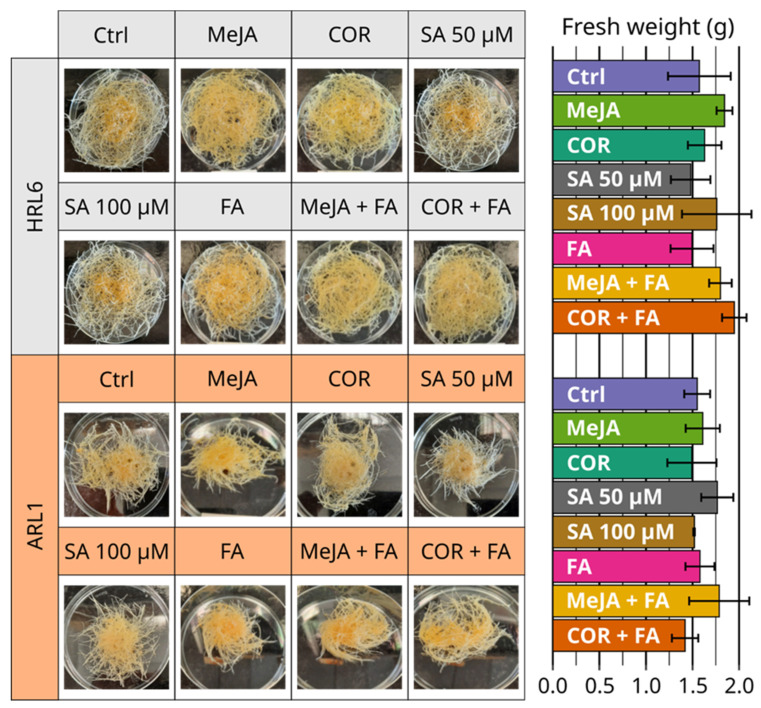
HRL6 and ARL1 cultures, control (Ctrl), and treated tissues with the elicitors and/or precursor feeding (**left panel**). Biomass accumulation of HRL6 and ARL1 expressed as FW (grams) in non-treated (Ctrl) and treated with MeJA, COR, SA, FA, MeJA + FA, and COR + FA (**right panel**). Each bar represents the average of three biological replicates ± SD.

To assess a possible inhibitory effect of the elicitors and/or precursor feeding on growth performance, the biomass of HRL6 and ARL1 were evaluated after each treatment. As reported in Figure 7, the FW achieved by both cultures after each treatment was comparable to the respective control. Indeed, the treatments with elicitors and/or precursor feeding did not affect the morphology and the color of both roots cultures (Figure 7).

**Figure 8:**
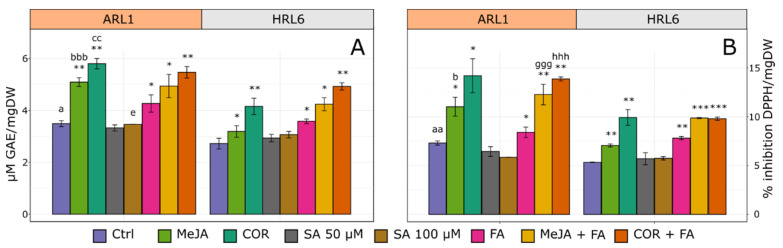
Total phenolic and radical scavenging activity in *Linum lewisii* root cultures non-treated (control) and treated with methyl jasmonate (MeJA), coronatine (COR), 50 µM (SA 50 µM), and 100 µM (SA 100 µM) salicylic acid, ferulic acid (FA), MeJA and FA (MeJA + FA) and COR and FA (COR + FA). (**A**) Total phenol content expressed as µg of gallic acid equivalent (GAE) per mg dry weight (DW). (**B**) Radical scavenging activity expressed as percentage of inhibition of 2,2-diphenyl-1-picrylhydrazyl (DPPH) per mg DW. Each bar represents the average of three biological replicates ± SD. Student’s t-test was applied: * indicates significance of treated samples versus control per each tissue (* *p* ≤ 0.05; ** *p* ≤ 0.01; *** *p* ≤ 0.001); apex letters indicate significance of the same treatment between the two tissue cultures, ^a^: control, ^b^: MeJA, ^c^: COR, ^d^: SA 50, ^e^: SA 100, ^f^: FA, ^g^: MeJA + FA, ^h^: COR + FA (^a^ *p* ≤ 0.05; ^aa^ *p* ≤ 0.01, ^aaa^ *p* ≤ 0.001).

To better characterize the two root cultures, the total phenols and the radical scavenging activity were evaluated after elicitors and precursor feeding treatments. As shown in Figure 8, COR and COR + FA treatments induced the highest accumulation of total phenols and the highest percentage of scavenging activity in both cultures. Conversely, the two SA concentrations (50 and 100 µM) had almost no effect either on HRL6 or ARL1. Specifically, the effect of COR elicitation on total phenol content expressed as µg gallic acid equivalent per mg DW (µg GAE/mgDW) was similar for ARL1 and HRL6 cultures being about 1.5 times higher than the respective control. However, the phenol content of non-treated ARL1 (Ctrl) was significantly higher than non-treated HRL6 (3.49 versus 2.72 µg GAE/mgDW); thus, the absolute amount of COR-treated ARL1 was significantly higher than HRL6 (5.80 ± 0.20 versus 4.15 ± 0.32 µg GAE/mgDW). Conversely, the effect of MeJA was more evident on ARL1 than on HRL6 cultures, increasing the ARL1 total phenol content about 1.5 times compared to its control (Figure 8A). The treatment with FA produced a significant increase in total phenols in both ARL1 and HRL6 compared to the control. However, when it was added in combination with MeJA or COR resulted significantly, with respect to MeJA or COR alone, only in HRL6 samples (*p* ≤ 0.05). To ensure that FA was absorbed by the root cultures, its concentration was determined in the medium at the end of the growing period. The HPLC analysis revealed the absence of FA in all the media tested (data not shown). The effect of elicitation was also evaluated on the antioxidant capacity of the extracts of the two different root cultures (Figure 8B). In all the samples analyzed, the free radical scavenging activity increased after elicitation with MeJA and COR. In particular, the trend observed for ARL1 and HRL6 subjected to all the elicitor treatments was similar to that described for total phenols. The higher antioxidant activities, expressed as a percentage of DPPH inhibition per mg DW, were shown by ARL1.

### 3.5. Effect of Elicitors on Justicidin B Accumulation

**Figure 9:**
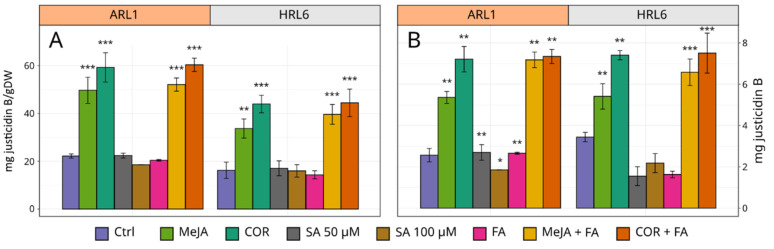
Accumulation of justicidin B in *Linum lewisii* root cultures extracts. (**A**) justicidin B expressed as mg/DW. (**B**) Justicidin B expressed as mg/flask. Each bar represents the average of three biological replicates ± SD. Student’s t-test was applied: * indicates significance of treated samples versus the respective control per each tissue (** *p* ≤ 0.01; *** *p* ≤ 0.001).

The effect of all the elicitor treatments on justicidin B accumulation in HRL and ARL root cultures was similar. The justicidin B amount was reported in Figure 9, and it was expressed both as mg/DW (Figure 9A) and as an absolute value (Figure 9B). It is important to evaluate both notations since the two root cultures’ growth was different, with HRL6 showing higher dry weight.

COR was the most effective elicitor treatment together with the combination of COR + FA. These treatments led to double the justicidin B content with respect to their controls. The treatment of the root cultures with SA had almost no effect on justicidin B production or even inhibited the accumulation of the molecule (Figure 9).

### 3.6. Cultivation of Roots Cultures in Bioreactor

**Figure 10:**
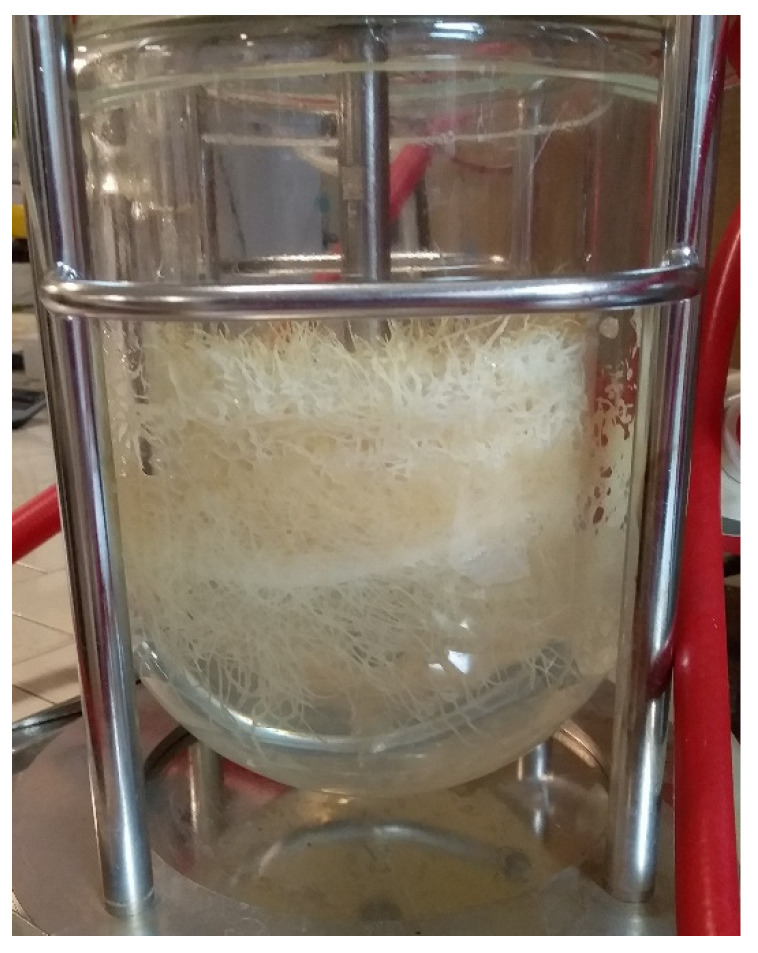
*Linum lewisii* HRL6 in bioreactor after four weeks of growth.

HRL6 and ARL1 lines were cultivated in a one-liter bioreactor with the aim of scaling up the production of justicidin B and analyzing the behavior of *L. lewisii* roots cultures in a bioreactor system. The cultures were grown in the bioreactor for three weeks and then elicited with MeJA and grown for a further one week. The respective controls were grown for four weeks. At the end of the experiment, the cultures did not show signs of aging (Figure 10).

As already observed in a flask (Figure 2 and Figure 7), the growth of the root cultures in the bioreactor and, therefore, the biomass reached has not been significantly affected by the elicitor treatment.

The amount of justicidin B in root extracts was determined at the end of the experiment/fermentation by HPLC. The justicidin B content (Table 1), expressed as the total amount reached by non-treated ARL1 and HRL6 after four weeks, was comparable (53.4 versus 49.3 mg). The treatment with MeJA more than doubled the justicidin B content in HRL6 and doubled in ARL1.

## 4. Discussion

In recent years, there has been a growing interest in secondary metabolites with pharmacological properties, with particular emphasis on lignans for their ability to prevent cancer and chronic diseases and for their antioxidant properties [46]. *Linum*, the largest genus of the family *Linaeceae*, has attracted considerable interest for its lignan content, including the most commonly known aryltetralin lignan such as podophyllotoxin and the arylnaphthalene lignan such as justicidin B. This last compound exhibits a wide array of biological activities ranging from piscicidal to antifungal, antiviral, antibacterial, and antioxidant. Justicidin B is also a cytotoxic substance on several cell lines, especially chronic myeloid and chronic lymphoid leukemia [20]. For these reasons, justicidin B is considered a potential lead compound for novel therapeutics.

Since approximately 80% of humans worldwide utilize medical plant material for primary health therapy, the exploitation of natural resources cannot meet increasing market needs [47]. Furthermore, artificial chemical synthesis is generally unsuitable in terms of economic costs. In this regard, in vitro cultures represent an interesting alternative approach to the production of bioactive compounds [48].

In this work, adventitious root and hairy root cultures were developed from *Linum lewisii*. The presence of justicidin B in *L. lewisii* cell cultures was highlighted by Konuklugil et al. [7], but to the best of our knowledge, no other studies have been conducted to analyze the production of this lignan from different tissue cultures of *L. lewisii*. Since suspension cell cultures show various constraints such as genetic instability, shear sensitivity, low yields, slow growth, and, most important, scale-up problems, in this work, we focused on root cultures that represent an alternative approach to the conventional method owing to their rapid growth, biosynthetic and genetic stability, and greater adaptability for large-scale production [49].

Considering biomass production, HRL performed better during six weeks of growth, raising 33% more biomass than ARL. The total phenol content and the antioxidant capacity expressed per mg of DW were slightly higher in ARL1 with respect to HRL6. However, the greater HRL6 biomass production largely compensates for the lower phenols and antioxidant activity of this tissue. Regarding the production of justicidin B, both ARL1 and HRL1 reached a peak of accumulation after five weeks of growth, and specifically, the hairy roots accumulated higher amounts of the molecule (+24%) with respect to ARL1. Konuklugil et al. reported that suspension cultures of *L. lewisii* produced justicidin B in the range of 0.16 to 0.30% of dry weight [7]. Conversely, the production of ARL and HRL reached 2.3% of tissue dry weight, thus confirming that differentiated tissues, ARL and HRL, represent a better source of phytochemicals than undifferentiated callus [50].

Elicitation has been extensively described as a strategy to enhance secondary metabolism. Culture treatments with molecules acting as environmental stress signaling, such as methyl jasmonate (MeJA), coronatine (COR), and salicylic acid (SA), have the ability to improve the production of secondary metabolites [27,51]. MeJA is a cyclopentanone compound that modulates a wide range of plant responses, and it has been reported to induce terpenoids, flavonoids, alkaloids, and phenylpropanoids [52]. COR, a toxin produced by the pathogen *Pseudomonas syringae,* is able to induce secondary metabolite biosynthesis even at lower concentrations than MeJA [51]. SA is the key signal regulating resistance to many pathogens and is able to elicit secondary metabolite production in plants [53]. The HRL6 and ARL1 lines were subjected to elicitation with MeJA, COR, and SA. The biomass growth was not significantly affected by the treatments. Different from what was previously observed for several plant tissues [41,51], elicitor treatment with MeJA, COR, and SA did not affect the growth of our cultures. Indeed, it has been reported that the same elicitor treatment can impact differently depending on the plant species [54,55]. Similar to what was reported for adventitious root and hairy root cultures of *L. austriacum* [41], MeJA and COR were effective in increasing total phenols and antioxidant activity. Specifically, ARL1 reached higher values for the two parameters. In particular, the increase in phenols and antioxidant activity induced by MeJA and COR was 1.5 and 1.9, respectively, for the two tissue cultures analyzed. COR was more effective than MeJA in both root tissues analyzed, reaching a level of 4.15 and 5.80 µg GAE/mg DW in HRL and ARL, respectively. Likewise to what observed in *L. thracicum* cell cultures treated with 50 µM and 100 µM SA [56], we found that elicitation of root cultures of *L. lewisii* with SA did not induced changes in the accumulation of phenol content. Indeed, the authors associated the elicitor treatment with browning of the culture and with a decrease in podophyllotoxin with respect to non-treated cell cultures [56]. This finding confirm that the elicitor effect is dependent on the plant species treated.

A strategy to further enhance the production of secondary metabolites in in vitro cultures is the simultaneous addition of elicitor and precursor to the growth medium [38]. Ferulic acid (FA), an early precursor of lignan intermediates, has been used in several tissue cultures [57]. Therefore, we performed elicitation experiments using FA in combination with MeJA or COR. Regarding the phenol content, the effect of FA alone on HRL6 and AR1 was almost negligible with respect to the control. The supply of FA conjugated with an elicitor compound (MeJA or COR) did not produce a significant increase in phenols in both tissues. The antioxidant activity after the combined elicitation was significantly higher in ARL1 than in HR6. However, there were no significant differences between the combined elicitation and the treatment with MeJA or COR alone within the same tissue extracts. These data suggest that both tissues could have already reached their maximum value after the MeJA and COR treatment alone.

In agreement with what we observed about total phenol content and with what was reported by Sasheva et al. in *L. thracicum* [56], the treatment of both root cultures with SA did not enhance the antioxidant capacity of the extracts.

Since the main lignan produced by *Linum* and *Dasylinum* sections is justicidin B [7,8], its accumulation has been followed in the two tissue cultures developed and in response to elicitor treatments. All the treatments tested significantly increased the amount of justicidin B in both root tissue cultures apart from SA and FA but did not alter the amount of justicidin B with respect to the controls. In particular, the amount of justicidin B reached in HRL and in ARL after different treatments was about two times that of the respective controls. The accumulation of justicidin B expressed as mg/gDW was higher in ARL1 than in HRL6 extracts. However, HRL6 cultures reached greater biomass than ARL1. Therefore, taking this difference into account and expressing the total amount of justicidin B per flask, the HRL6 production of justicidin B resulted in 28% higher. This result accurately reflects the difference in biomass weight reached by the two different tissue cultures. As far as the elicitation with MeJA or COR in combination with FA, no significant differences were observed with respect to the elicitation with MeJA or COR alone, even if FA was successfully absorbed from the medium. As already mentioned, this result suggests that the tissues could have reached an accumulation threshold value of justicidin B.

The production of justicidin B obtained from *L. lewisii* HRL and ARL cultures was significantly greater than that reported for *L. austriacum* root cultures by Mascheretti et al. [41] and by Mohagheghzadeh et al. [11]. Even the amount of justicidin B reached by hairy roots of *L. narbonense*, which belongs to the same clade of *L. lewisii* and *L. austriacum*, was lower than the content reached by our tissues. The results obtained highlight a better performance of HRL6 in terms of biomass accumulation, fast growth rate, genetic and biochemical stability, relatively simple maintenance in phytohormone-free media, and more lateral branching.

In addition, HRL6 and ARL1 were tested in a pilot scale-up experiment using a small bioreactor (1 L). Both lines showed a suitable adaptation to the new environment and confirmed the data obtained in a flask. In particular, HRL6 elicited with MeJA resulted in more productive tissue (Table 1).

Besides justicidin B, other derivatives of this compound were highlighted in the root tissue cultures, isojusticidin and 7-*O*-β-d-apiofuranosyl-diphyllin (tuberculatin), identified in *L. lewisii* for the first time. In addition, 7-*O*-β-d-xylofuranosyl-(1→5)-*O*-β-d-xylofuranosyl-diphyllin and 7-*O*-β-d-xylofuranosyl-(1→5)-*O*-β-d-xylofuranosyl-(1→5)-*O*-β-d-glucosyl-diphyllin were identified as new molecules. Tuberculatin and diphyllin have been described for their cytotoxic effects against several cancer cells in vitro [58]. In particular, it has been reported that glycosylated diphillins are endowed with relevant antiviral activities against SARS-CoV-2 [59], and particularly 6-deoxyglucose-diphyllin (DGP) against Zika virus [60]. Moreover, it has been reported that nearly 1200 secondary metabolites containing apiose, including various phenolics (e.g., phenylpropanoids, lignans, flavonoids), have been identified in several plant species. Usually, the synthesis of glycosylated defense compounds represents a strategy to store bioactive molecules in the inactive form [61,62].

## 5. Conclusions

Plant cell and tissue cultures represent a suitable strategy for the production of useful biochemicals. Lignans are a class of phenolic compounds showing a wide spectrum of biological properties that have been gaining interest in the last years. In this work, adventitious and hairy roots were developed from *Linum lewisii* for the production of the arylnaphthalene lignan justicidin B, the assessment of total phenol content, and the antioxidant activity and the effect of several elicitors. Both the tissues developed demonstrated a very suitable growth rate, with HRL showing the higher biomass accumulation. In addition, the justicidin B production followed the trend of biomass. Among the different elicitors used, MeJA and COR were the most effective. Namely, after MeJA treatment, justicidin B doubled in ARL and increased by two and a half times in HRL, whereas phenols and antioxidant activity were doubled. Besides justicidin B, other derivatives were highlighted in the root tissue cultures: isojusticidin, tuberculatin, and two glycosylated diphyllin compounds. These last two compounds were identified as new molecules. The ability of ARL and HRL cultures to grow in a small bioreactor was reported suggesting a better adaptation of HRLs with respect to ARLs.

These results would be useful to enhance the production of bioactive compounds from *L. lewisii* and would also be applied to other plant cell or tissue cultures.

## Figures and Tables

**Table 1:** Justicidin B production by HRL6 and ARL1 in bioreactor after four weeks expressed as total amount (mg) and as concentration (mg/L).

		Justicidin B	
		mg	mg/L
ARL1	Ctrl	49.3	75.8
	MeJA	99.2	152.7
HRL6	Ctrl	53.4	82.2
	MeJA	132.6	204.1

## Data Availability

The data is contained within the article or Supplementary Material.

## Supplementary Materials

The following supporting information can be downloaded at: https://www.mdpi.com/article/10.3390/antiox11081526/s1, Figure S1: COSY-DQF of 7-*O*-β-d-xylofuranosyl-(1→5)-*O*-β-d-xylofuranosyl-diphyllin; Figure S2: ^13^C DEPT-135 of 7-*O*-β-d-xylofuranosyl-(1→5)-*O*-β-d-xylofuranosyl-diphyllin; Figure S3: ^1^H–^13^C edited HSQC of 7-*O*-β-d-xylofuranosyl-(1→5)-*O*-β-d-xylofuranosyl-diphyllin; Figure S4: ^1^H–^13^C HMBC of 7-*O*-β-d-xylofuranosyl-(1→5)-*O*-β-d-xylofuranosyl-diphyllin; Figure S5: COSY-DQF of 7-*O*-β-d-xylofuranosyl-(1→5)-*O*-β-d-xylofuranosyl-(1→5)-*O*-β-d-glucosyl-diphyllin; Figure S6: ^13^C DEPT-135 of 7-*O*-β-d-xylofuranosyl-(1→5)-*O*-β-d-xylofuranosyl-(1→5)-*O*-β-d-glucosyl-diphyllin; Figure S7: ^1^H–^13^C edited HSQC of 7-*O*-β-d-xylofuranosyl-(1→5)-*O*-β-d-xylofuranosyl-(1→5)-*O*-β-d-glucosyl-diphyllin; Figure S8: ^1^H–^13^C HMBC of 7-*O*-β-d-xylofuranosyl-(1→5)-*O*-β-d-xylofuranosyl-(1→5)-*O*-β-d-glucosyl-diphyllin.
