# Supplementary material for: Linum lewisii Adventitious and Hairy-Roots Cultures as Lignan Plant Factories
Source: Antioxidants (Basel). 2022 Aug 5;11(8):1526. doi: 10.3390/antiox11081526 (PMC9404846; doi:10.3390/antiox11081526)
Supplement: Supplementary file 1 [file antioxidants-11-01526-s001.zip › antioxidants-1837781-supplementary.pdf]

Linum lewisii adventitious and hairy-roots cultures as lignan plant factories

Supplementary Materials

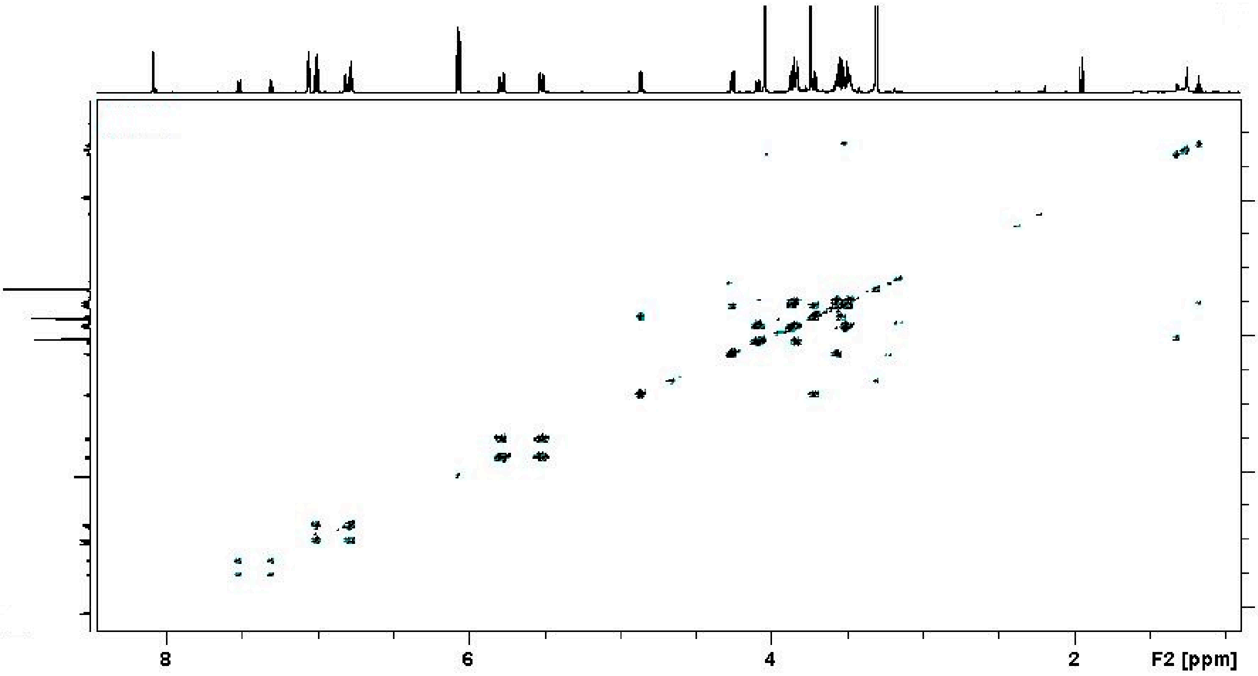

Figure S1. COSY-DQF of 7-O-β-D-xylofuranosyl-(1→5)-O-β-D-xylofuranosyl-diphyllin.

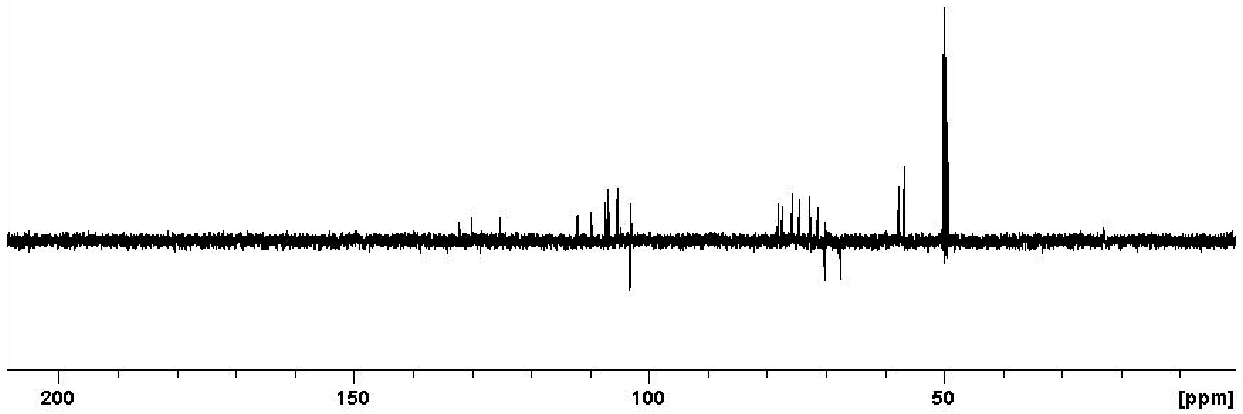

Figure S2. ^{13}C DEPT-135 of 7-O-β-D-xylofuranosyl-(1→5)-O-β-D-xylofuranosyl-diphyllin.

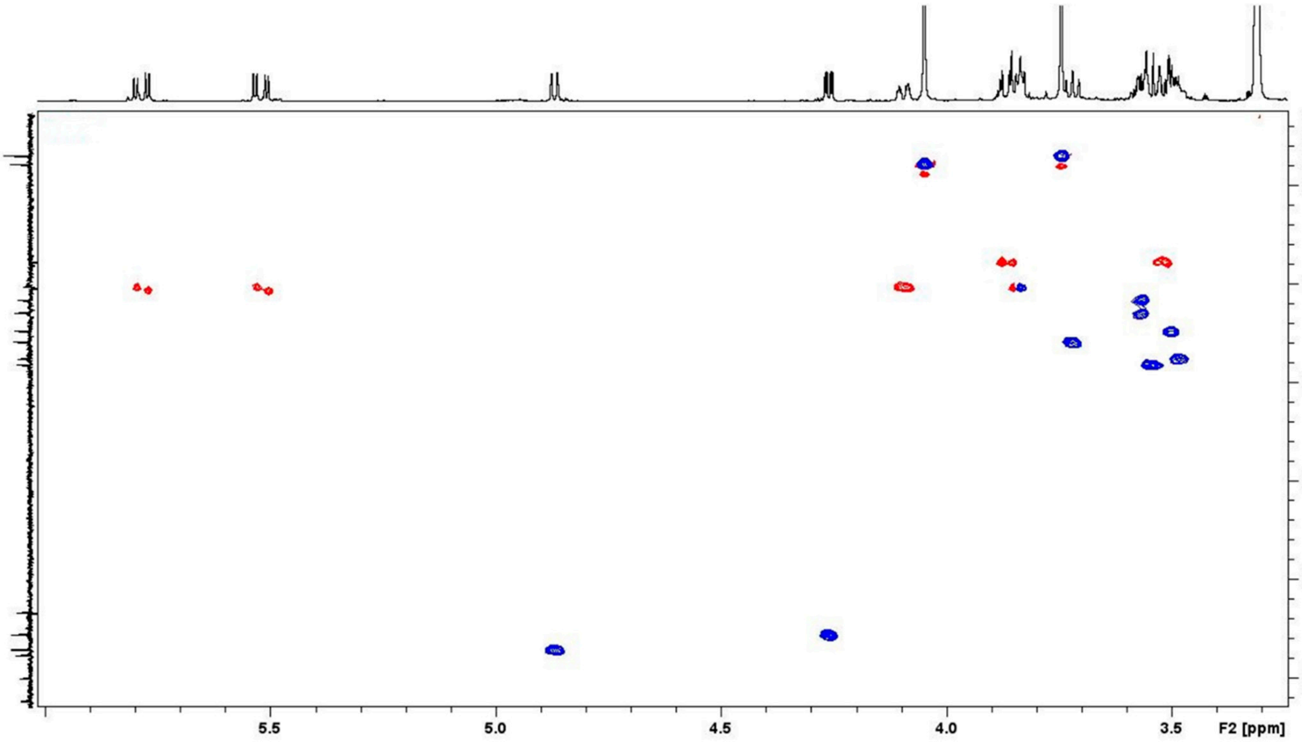

Figure S3. ^{1}H-^{13}C edited HSQC of 7-O-β-D-xylofuranosyl-(1→5)-O-β-D-xylofuranosyl-diphyllin.

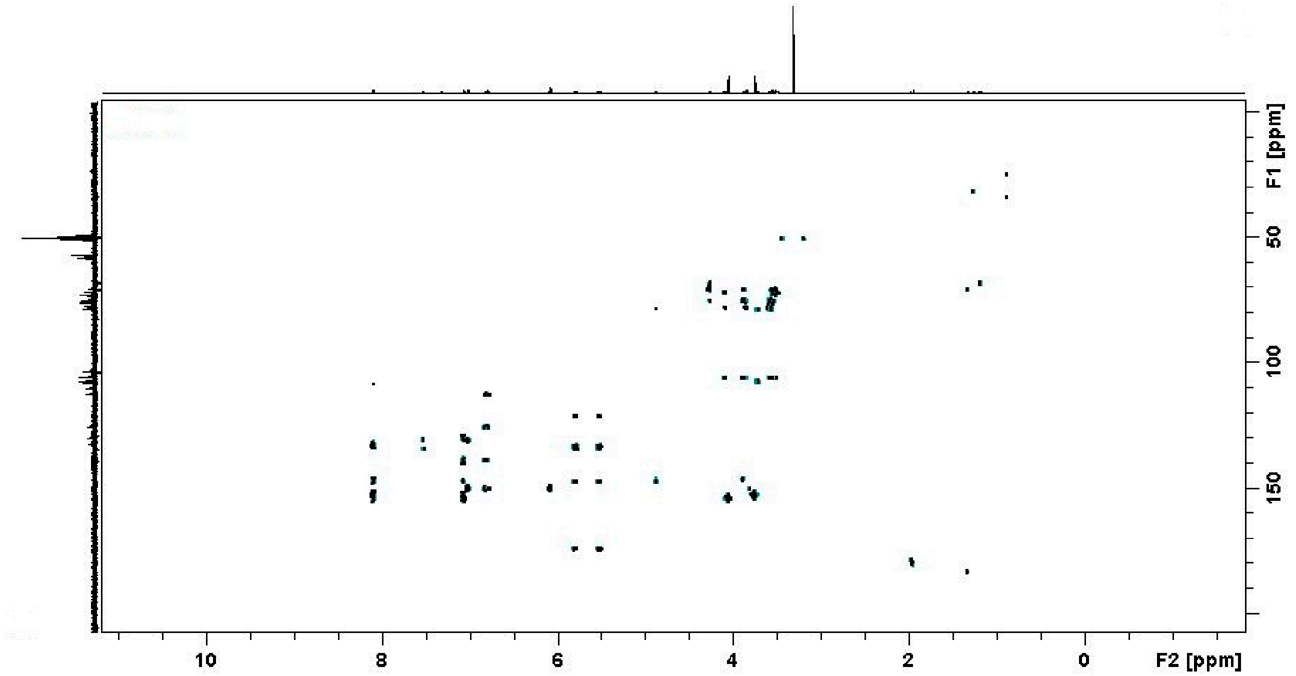

Figure S4. ^{1}H-^{13}C HMBC of 7-O-β-D-xylofuranosyl-(1→5)-O-β-D-xylofuranosyl-diphyllin.

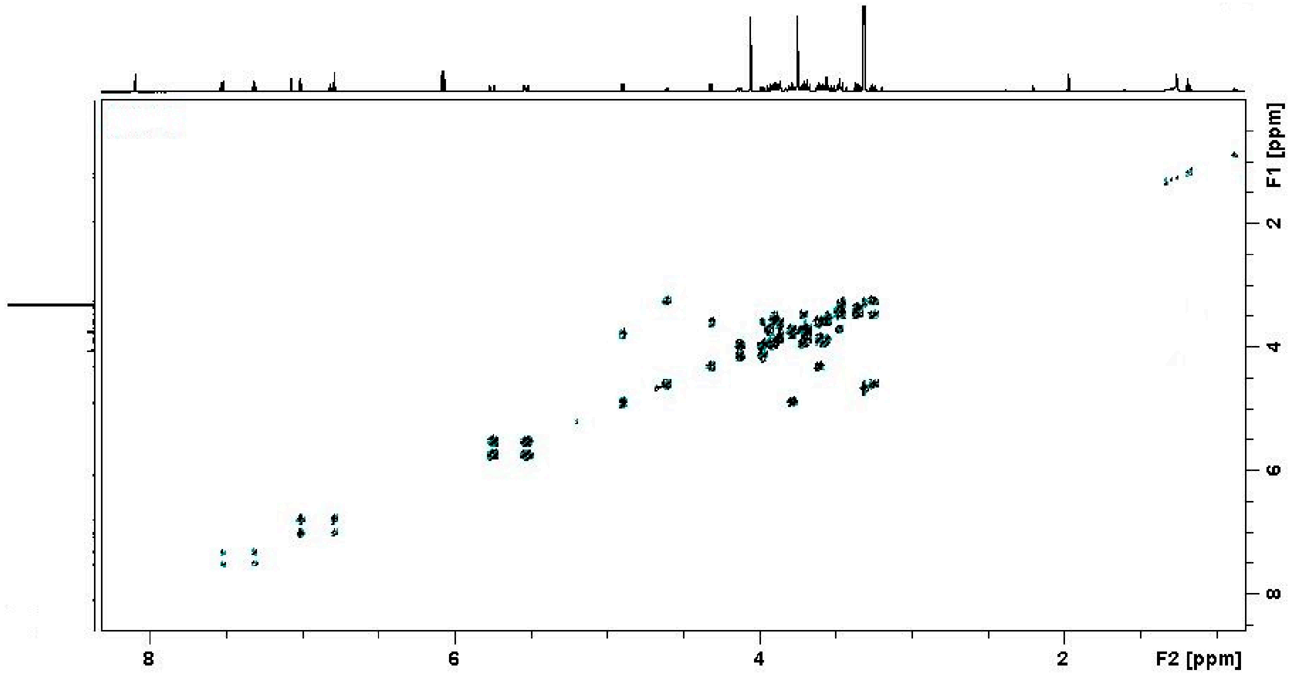

Figure S5. COSY-DQF of 7-O-β-D-xylofuranosyl-(1→5)-O-β-D-xylofuranosyl-(1→5)-O-β-D-glucosyl-diphyllin.

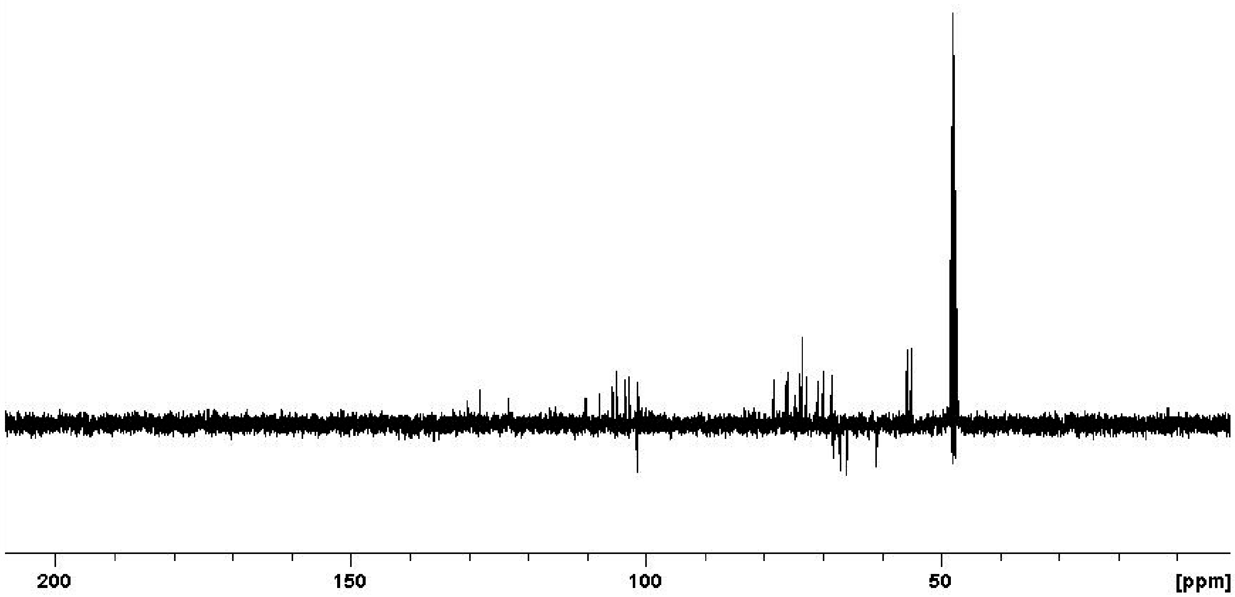

Figure S6. ^{13}C DEPT-135 of 7-O-β-D-xylofuranosyl-(1→5)-O-β-D-xylofuranosyl-(1→5)-O-β-D-glucosyl-diphyllin.

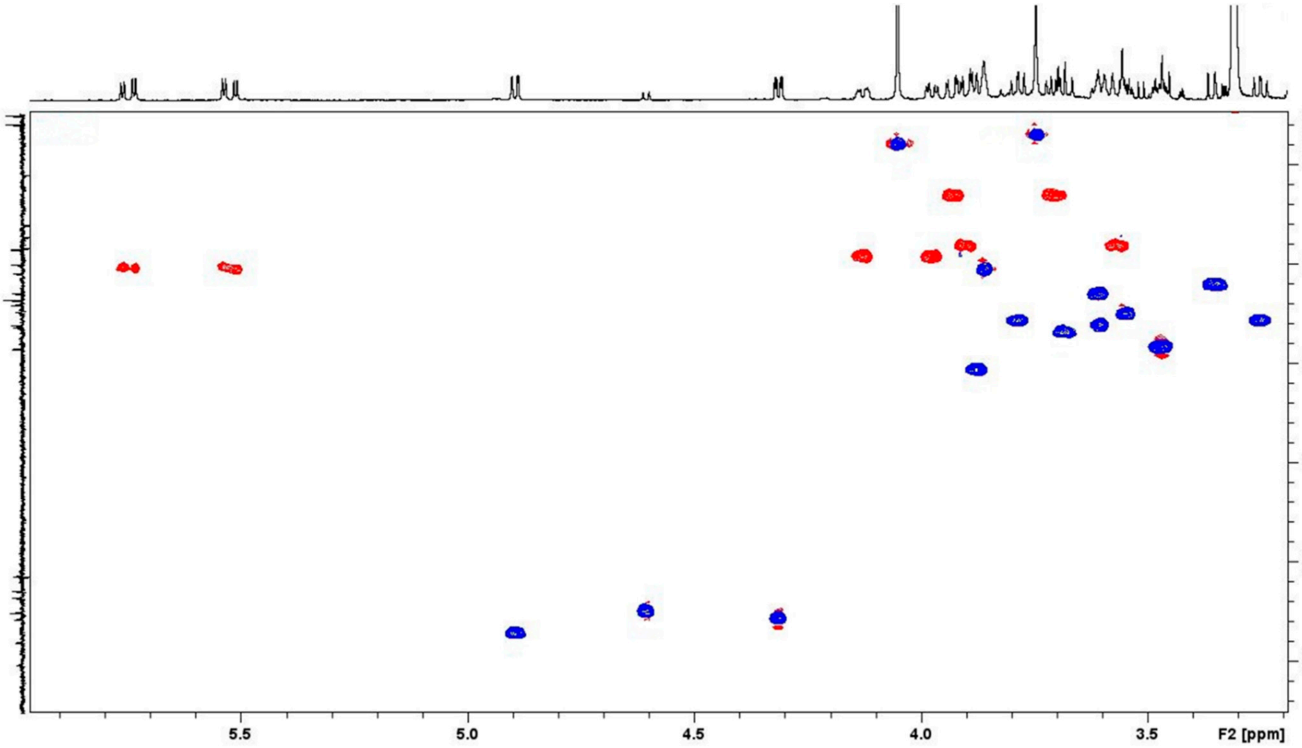

Figure S7. ^{1}H-^{13}C edited HSQC of 7-O-β-D-xylofuranosyl-(1→5)-O-β-D-xylofuranosyl-(1→5)-O-β-D-glucosyl-diphyllin.

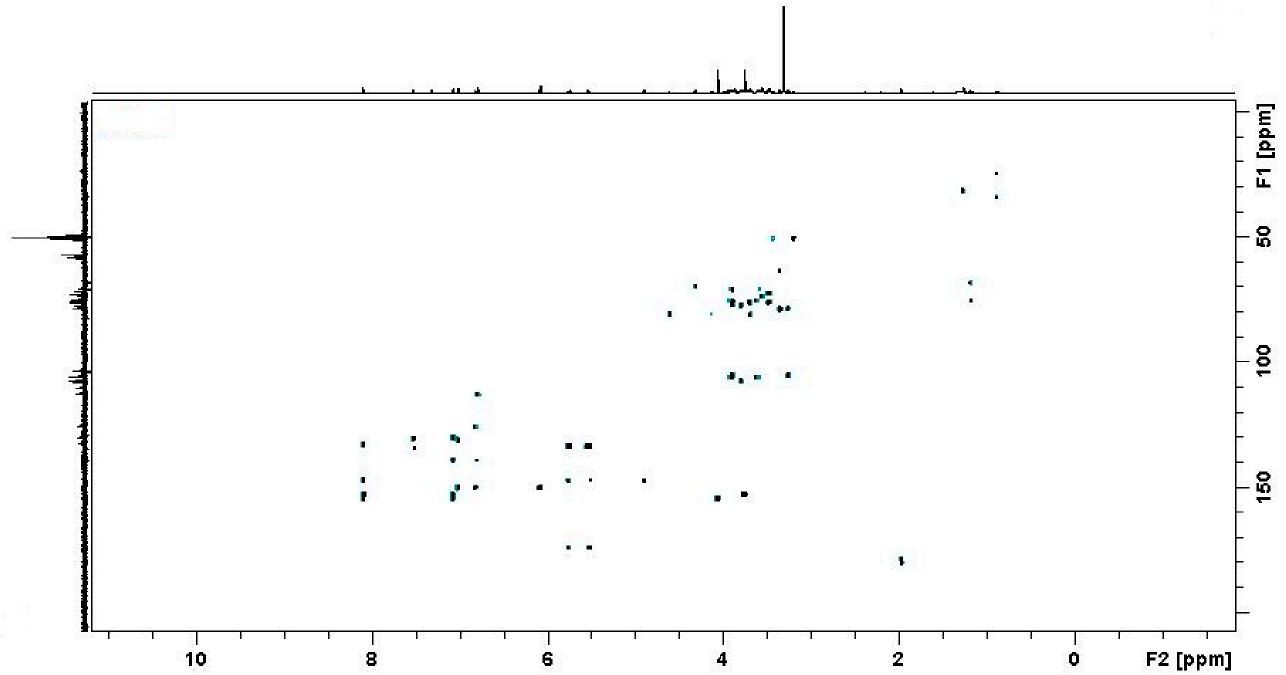

Figure S8. ^{1}H-^{13}C HMBC of 7-O-β-D-xylofuranosyl-(1→5)-O-β-D-xylofuranosyl-(1→5)-O-β-D-glucosyl-diphyllin.
